# A pediatric case of endoscopic fistula closure using a polyglycolic acid sheet

**DOI:** 10.1055/a-2095-2165

**Published:** 2023-06-22

**Authors:** Mitsuhiro Kono, Yasuaki Nagami, Tatsuo Nakaoka, Akifumi Matsuki, Masaki Ominami, Shusei Fukunaga, Yasuhiro Fujiwara

**Affiliations:** 1 Department of Gastroenterology, Osaka Metropolitan University Graduate School of Medicine, Osaka, Japan; 2 Department of Surgical Medicine, Osaka Metropolitan University Graduate School of Medicine, Osaka, Japan

Recurrent tracheoesophageal fistula is a common postoperative complication of esophageal atresia 1 . The surgical treatments utilize muscle and pleural flaps 2 . Furthermore, endoscopic fistula closure with fibrin glue, a biomaterial, can be achieved by epithelializing, promoting circulation, and inhibiting leukocyte infiltration 1 . In adults, endoscopic fistula closure using a polyglycolic acid (PGA) sheet is useful for treating postoperative esophageal anastomotic fistulas 3 . However, no such pediatric reports are available.

Herein, we report the first pediatric case of endoscopic fistula closure with PGA sheet ( Video 1 ).

**Video 1** Use of polyglycolic acid sheets and fibrin glue as an effective alternative to standard procedures during pediatric endoscopic fistula closure.


A 3-year-old girl was admitted with the chief complaint of persistent fever and cough after eating. She underwent postnatal thoracoscopic radical esophagectomy for type C esophageal atresia. Owing to postoperative complication, she underwent multiple endoscopic balloon dilations.

Esophagogastroduodenoscopy revealed an esophageal fistula on the oral posterior wall of the esophageal anastomosis ( Fig. 1 ). Tracheal esophagography ( Fig. 2 ) revealed tracheoesophageal fistula.

**Fig. 1:**
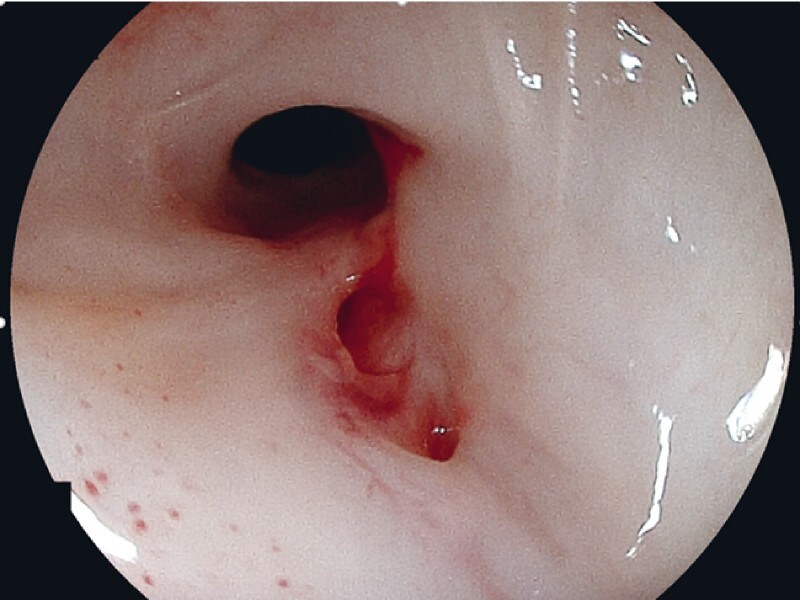
A fistula on the posterior wall of the oral side of the esophageal anastomosis.

**Fig. 2:**
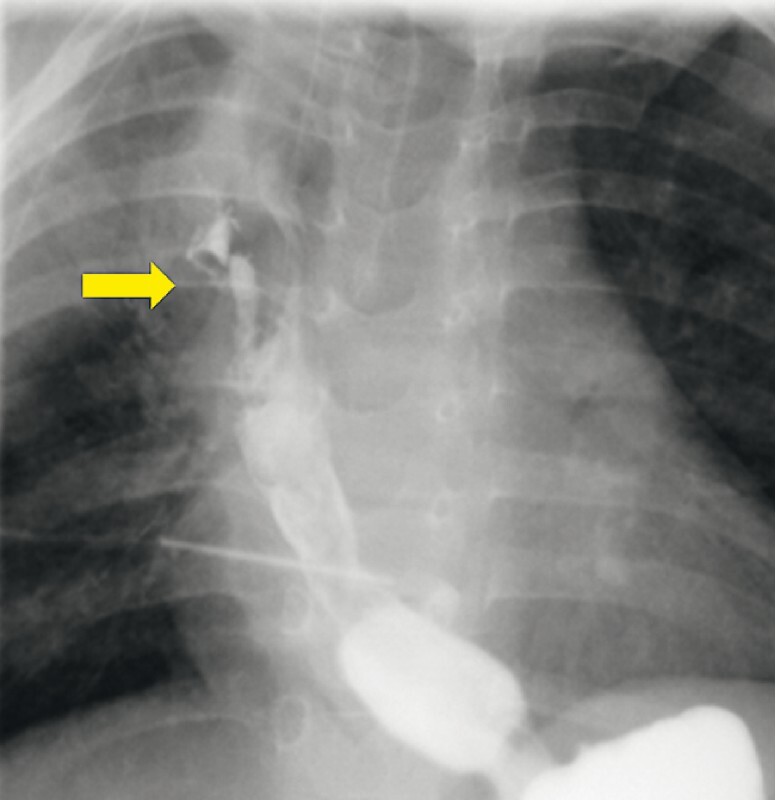
Esophagography revealed the trachea (yellow arrow).

First, mucosa around the fistula was cauterized using hot biopsy through a single-channel upper gastrointestinal endoscope ( Fig. 3 ). Subsequently, small pieces of PGA sheet (Neoveil; Gunze Co., Osaka, Japan) were grasped with biopsy forceps, immersed in fibrinogen solution, and used to fill the fistula through the scope ( Video 1 ). Finally, fibrinogen and thrombin solutions of fibrin glue (Beriplast P Combi-Set; CSL Behring Pharma, Tokyo, Japan) were applied to the PGA sheets ( Fig. 4 ).

**Fig. 3:**
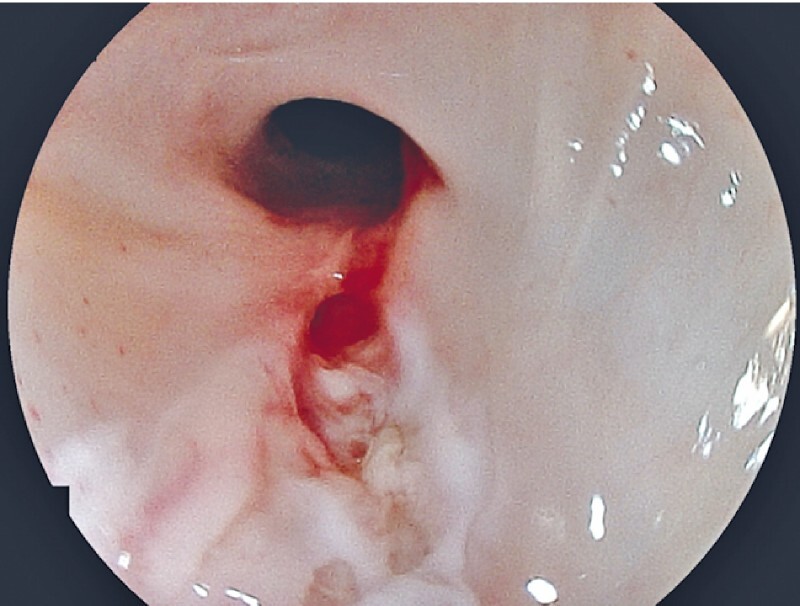
Image after cauterization of the area around the fistula with hot biopsy forceps, soft mode 80 W of electrosurgical generator (VIO300D; ERBE Elektromedizin GmbH, Tübingen, Germany).

**Fig. 4:**
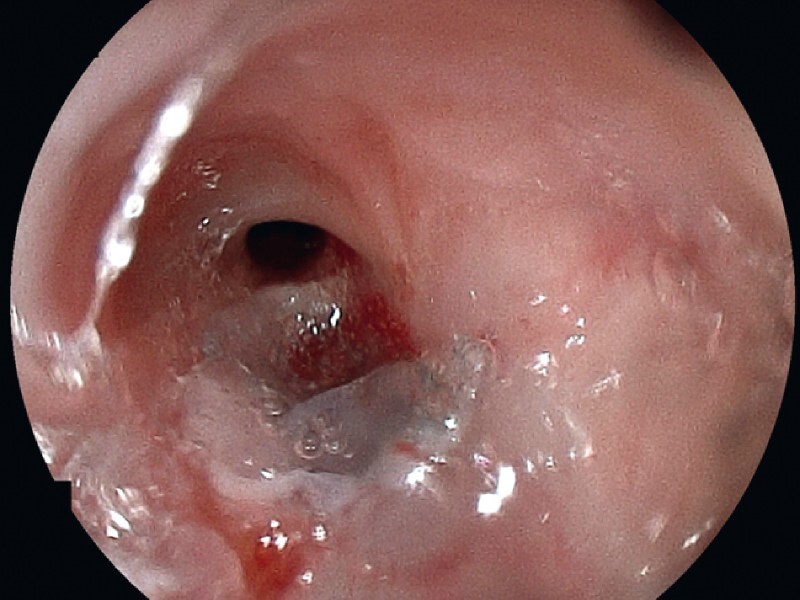
Fibrinogen and thrombin solutions of fibrin glue were applied to the polyglycolic acid sheets.

Endoscopy 3 weeks later confirmed fistula closure ( Fig. 5 ), and oral intake by the patient was possible without symptoms.

**Fig. 5:**
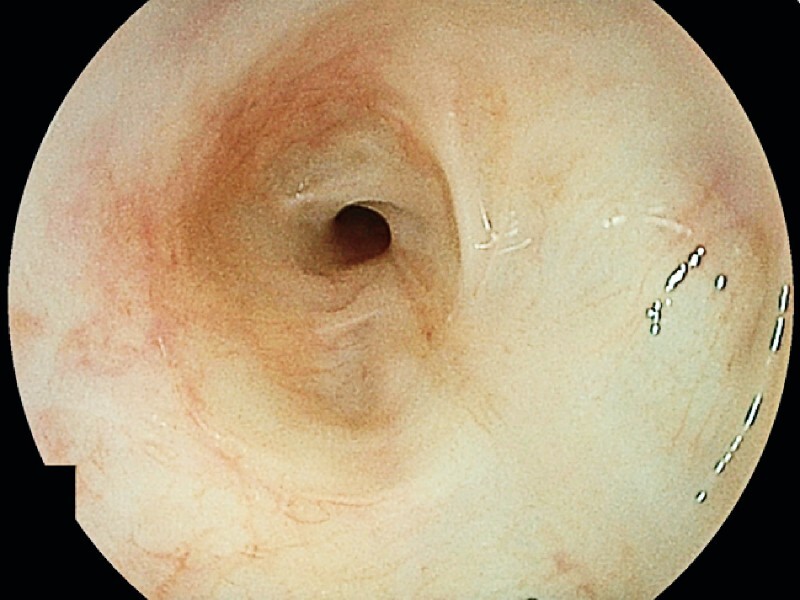
Endoscopy after 3 weeks revealed closure of the fistula.

Fibrin glue is generally used in the treatment of pediatric tracheoesophageal fistula. PGA sheets acting as tissue-regenerative scaffolds may effectively help in the healing process, as granulation tissue can fill and cover the fistula 3 .

Endoscopy_UCTN_Code_TTT_1AO_2AI

## Notes

Endoscopy E-Videos

https://eref.thieme.de/e-videos
*E-Videos* is an open access online section of the journal *Endoscopy* , reporting on interesting cases and new techniques in gastroenterological endoscopy. All papers include a high-quality video and are published with a Creative Commons CC-BY license. Endoscopy E-Videos qualify for HINARI discounts and waivers and eligibility is automatically checked during the submission process. We grant 100% waivers to articles whose corresponding authors are based in Group A countries and 50% waivers to those who are based in Group B countries as classified by Research4Life (see: https://www.research4life.org/access/eligibility/ ). This section has its own submission website at https://mc.manuscriptcentral.com/e-videos
